# C-type allatostatins mimic stress-related effects of alarm pheromone on honey bee learning and memory recall

**DOI:** 10.1371/journal.pone.0174321

**Published:** 2017-03-21

**Authors:** Elodie Urlacher, Jean-Marc Devaud, Alison R. Mercer

**Affiliations:** 1 University of Otago, Department of Zoology, Dunedin, New Zealand; 2 Centre National de la Recherche Scientifique (CNRS), Centre de Recherches sur la Cognition Animale (UMR 5169), Toulouse, France; 3 Université de Toulouse, UPS Centre de Recherches sur la Cognition Animale (UMR 5169), Toulouse, France; Biocenter, Universität Würzburg, GERMANY

## Abstract

As honey bee populations worldwide are declining there is an urgent need for a deeper understanding of stress reactivity in these important insects. Our data indicate that stress responses in bees (*Apis mellifera* L.) may be mediated by neuropeptides identified, on the basis of sequence similarities, as allatostatins (ASTA, ASTC and ASTCC). Effects of allatostatin injection are compared with stress-related changes in learning performance induced by the honeybee alarm pheromone, isopentylacetate (IPA). We find that bees can exhibit two markedly different responses to IPA, with opposing effects on learning behaviour and memory generalisation, and that strikingly similar responses can be elicited by allatostatins, in particular ASTCC. These findings lend support to the hypothesis that allatostatins mediate stress reactivity in honey bees and suggest responses to stress in these insects are state dependent.

## Introduction

When honey bee workers guarding a colony perceive a threat, they release alarm pheromones from their sting apparatus that induce stress-like responses in their nestmates [1–5]. These responses include a slowing of the rate at which bees learn to associate odours with a food reward [6–9]. Parallels can be observed in rats, which show a lowering of appetitive-related behaviours in response to stress [10]. Alarm pheromone also induces opioid-like analgesia in bees [3,5], as it does in mammals [11], and treating bees with the vertebrate opioid-receptor agonist, morphine, decreases nociception [5], as well as appetitive learning [12–13]. However, whether an endogenous opioid system exists in insects is still debated because in insects, opioid-like peptides have yet to be identified [14].

At the receptor level, similarities have been identified between opioid and closely related systems in mammals and allatostatin (AST) systems in insects [14–16]. Allatostatins (ASTs), as their name suggests, were originally identified as neuropeptides that inhibit the biosynthesis of juvenile hormone (JH) in the *corpora allata* of insects [reviewed by 17]. Three structurally distinct subfamilies of neuropeptides (A-, B- and C-type ASTs), were identified that share this function [17]. However, the name ‘allatostatin’ can be misleading, as the allatostatic properties of these neuropeptides are not shared across all insect groups [17]. Many putative ASTs, including the honey bee (*Apis mellifera*) neuropeptides examined in this study, have been identified based on sequence similarity rather than biological activity. At least 5 neuropeptides categorized as A-type allatostatins are expressed in the brain of the bee [18–20], all of which share the Y/FXFGL-NH2 consensus sequence characteristic of this subfamily [17,21]. The most abundant A-type AST in the brain, and the form examined in this study, is ASTA4 (GRQPYSFGL-amide) [18]. As yet, no gene encoding for peptides that fall into the B-type category of ASTs has been identified in honey bees, but two paralogue genes encoding C-type peptides have been identified; Apime-ASTC (SYWKQCAFNAVSCF-amide) and Apime-ASTCC (GQAKGRVYWRCYFNAVTCF) [20]. As both lack the consensus C-terminal PISCF sequence typical of the C subfamily [20], they are referred to here as ASTC and ASTCC respectively, instead of PISCF/ASTs as recommended by [22].

Two AST receptor genes in *Apis mellifera* have been cloned and the proteins characterised, *Apime*-ASTA-R and *Apime*-ASTC-R. *Apime*-ASTA-R responds to the A-type AST, *Apime*-ASTA, whereas *Apime*-ASTC-R is activated by C-type ASTs, *Apime*-ASTC and *Apime*-ASTCC [16]. While both receptors were originally identified based on their similarity to opioid receptors, insect ASTA receptors are most closely related to galanin receptors in vertebrates, whereas ASTC receptors are more closely related to vertebrate somatostatin receptors [14–17]. Opioids, somatostatin and galanin are among many neuropeptides in the vertebrate brain that not only influence learning and memory [23], but participate also in stress physiology [24–25]. ASTA, ASTC and ASTCC, and the receptors that mediate their actions, are expressed throughout the brain of the bee [16,19,26], including in centres involved in olfactory-information processing and associative learning, such as the antennal lobes and mushroom bodies of the brain [27]. While allatostatins are generally described as inhibitory neuropeptides [20,28], their role(s) in the insect brain remain unclear. When injected into the brain of the bee, all three honey bee ASTs (A, C and CC) modulate appetitive learning behaviour [16]. Interestingly, their effects on learning are reminiscent of the stress-inducing effects of alarm pheromone [6–8], suggesting that in bees ASTs may play a role in mediating physiological and behavioural responses to stress. Consistent with this possibility, ASTs are known to regulate the synthesis of juvenile hormone (JH) in some insect groups [17], a hormone that has been linked to stress reactivity in bees [29–31].

As honey bees show remarkably fast and robust learning abilities in laboratory settings [32–35], stress-induced changes in appetitive learning and memory recall provide a useful model for studying stress reactivity and its underlying physiological mechanisms. Here we use this model to examine the modulatory actions of honey bee ASTs on learning behaviour and their potential role as mediators of alarm pheromone-induced stress responses.

## Results

### Effects of IPA and ASTs on acquisition rate and 1-hour memory recall

**Fig 1:**
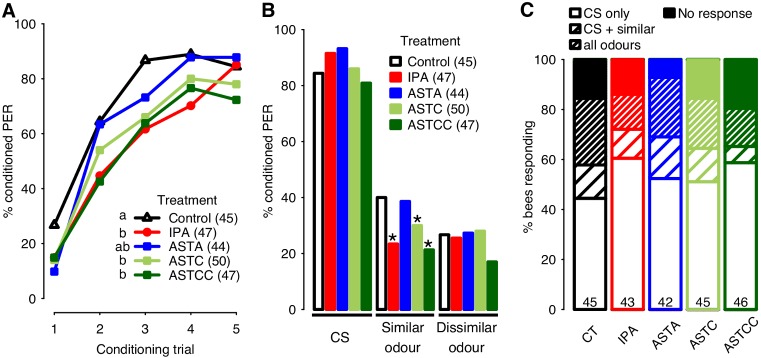
Type 1 responses: Reduced acquisition rate and lower levels of generalisation. (**A**) Acquisition curves show changes in the percentages of forager bees displaying the conditioned proboscis extension response (PER) over five successive conditioning trials. Inset: key to the 5 groups tested. Letters (a, b) indicate significant differences between groups (refer to text for p values); groups that share a letter are not significantly different. The number of bees in each group is indicated in parentheses. (**B**) Results of the 1-hour memory test. Response levels across the 5 groups tested do not differ for either the conditioned stimulus (CS, 1-nonanol) or for the dissimilar odour (2-hexanol). However, in IPA-, ASTC- and ASTCC-treated bees the level of responses to the similar odour (nonanal) is significantly lower than the level recorded in controls (refer to text for p values, indicated by an asterisk). (**C**) Categorisation of responses during the memory test. No significant differences were detected between the distributions of control (CT), IPA- or AST-treated bees into the main different response categories (CS alone, CS plus the similar odour, all 3 odours, none of the odours tested). The number of bees in each group is indicated at the bottom of each bar.

Foragers were either exposed to the main component of alarm pheromone, isopentyl acetate (IPA), or injected with ASTA, ASTC or ASTCC one hour before conditioning. Overall, the percentages of bees displaying conditioned proboscis extension responses (conditioned PER) to an odour associated with a sucrose reward increased significantly across the 5 conditioning trials (1.96±0.13, p<0.001), demonstrating effective learning in all groups (Fig 1A). However, learning performance was affected by treatment. Bees exposed to IPA displayed lower levels of conditioned PER than controls (-1.27±0.50, p<0.05, Fig 1A). This was true also of bees treated with ASTC or ASTCC (-0.97±0.48, p<0.05 and -1.33±0.51, p<0.01, respectively).

To explore further the similarities and differences between the effects of IPA and ASTs, we next compared the 1-hour memory performances of bees by presenting odours without reward. Most bees responded to the conditioned stimulus (CS) and no differences in the levels of responses to this odour were identified between groups (Fig 1B). Moreover, in all groups, bees responded more to the CS than to either of two non-reinforced odours (p<0.001 in all). However, in IPA-exposed bees and in bees treated with ASTC or ASTCC the percentages of bees responding to the "similar odour", chosen for its perceptual similarity to the CS [36], were significantly lower than in controls (-3.14±1.22, p<0.05 for IPA; -2.30±1.16, p<0.05 for ASTC; -3.10±1.31, p<0.05 for ASTCC). To determine whether the reduction in responses to the similar odour in these groups was accompanied by an increase in the level of responses to the conditioned stimulus alone (increased CS specificity), we examined the response distributions of individual bees within each group (Fig 1C). IPA- and ASTCC-treated bees, which displayed the lowest levels of generalisation (responses to odours other than CS), also displayed the highest levels of responses to the CS alone (60% and 59%, respectively). However, the distribution of responses within the 4 response categories did not differ significantly across the five groups (χ^2^ = 9.26, df = 12, p = 0.68).

In all groups, the level of ‘spontaneous’ responses to the CS in the first conditioning trial was relatively high (Fig 1A). Excluding such bees from the dataset (S1 Fig) did not alter the identification of slower acquisition rates in IPA- and ASTCC-treated bees. Trends suggesting enhanced response specificity in the memory tests were also clearly apparent, although effects on memory recall were no longer statistically significant.

### A shift to enhanced generalisation (Type 2 responses)

**Fig 2:**
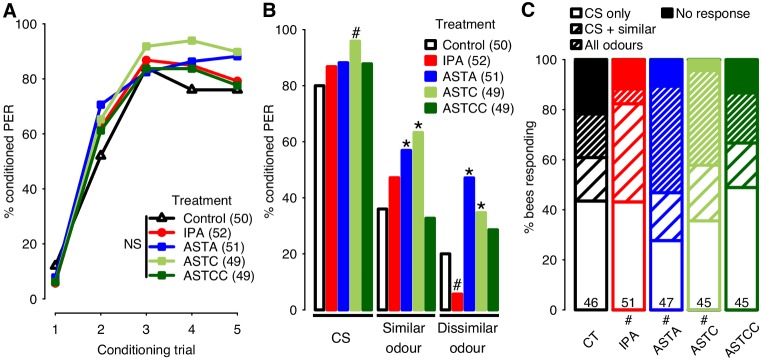
Type 2 responses: Enhanced generalisation. (**A**) Acquisition curves show changes in the percentage of forager bees displaying the conditioned proboscis extension response (PER) over five successive conditioning trials. Inset: key to the 5 groups tested. The number of bees in each group is indicated in parentheses. No significant differences were detected (NS). (**B**) Results of the 1-hour memory test. The percentages of bees responding to the CS (1-nonanol) were slightly higher in treated bees than in controls, particularly in ASTC-treated bees (#, p<0.1). The levels of responses to the similar odour, and to the dissimilar odour were significantly higher in ASTA- and ASTC-treated bees than in controls (asterisks, refer to text for p values). (**C**) Categorisation of responses in the memory test. The percentage of bees responding to all 3 odours was highest in ASTA-treated bees, and high also in bees treated with ASTC. IPA-treated bees showed lower levels of generalisation, mostly to the similar odour but less to the dissimilar odour. While a significant difference was detected between the response distributions of the different groups, comparisons of each treatment versus control only revealed trends (# 0.0125<p<0.05, alpha = 0.0125 after Bonferroni correction). The number of bees in each group is indicated at the bottom of each bar.

Typically, animals responding to IPA, ASTC or ASTCC display a slower acquisition rate than controls (Fig 1A, [6–8]). In the 1-hour memory test, however, IPA-, ASTC- or ASTCC-treated bees generally responded less than controls to an odour perceptually similar to the CS (Fig 1B), suggesting that memory specificity is enhanced. However, during the course of this study, we observed short periods of time (typically lasting less than a month) when IPA exposure and treatment with allatostatins induced effects on behaviour that differed markedly from those described above (compare Figs 1 and 2). At such times, learning, as evidenced by increasing percentages of conditioned responses over successive conditioning trials, was still clearly apparent in all of the groups tested (2.35±0.16, p<0.001, Fig 2A), but in contrast to expectation, acquisition rate was not negatively affected by exposure to IPA (0.06±0.36, p = 0.88), nor by treatments with ASTA (0.19±0.36, p = 0.59), ASTC (0.11±0.37, p = 0.76) or ASTCC (0.12±0.37, p = 0.75).

During these periods, responses in the 1-hour memory test were also strikingly atypical. While bees responded more to the CS than to the other two odours tested, as expected (p<0.001 in all cases), the responsiveness of bees treated with IPA or allatostatin was *higher* overall than in controls (Fig 2B). For example, bees treated with ASTC tended to respond more to the CS than controls (2.53±1.32, p = 0.06), and the percentage of bees responding to the similar odour was significantly higher in ASTA- and ASTC-treated bees than in controls (2.07±0.70, p<0.01 and 1.87±0.70, p<0.01, respectively). Both groups also responded more strongly than controls to the dissimilar odour (1.96±0.73, p<0.01 and 1.71±0.74, p<0.05), although interestingly, responses to the dissimilar odour tended to be lower than expected in bees exposed to IPA (1.81±0.98, p = 0.06).

Enhanced odour generalisation in bees treated with IPA, ASTA and ASTC has not been described previously. For simplicity, we refer to such responses as “Type 2” responses (Fig 2) and to the more typical responses induced by IPA and ASTs as “Type 1” responses (Fig 1). At times when bees were displaying Type 2 responses, enhanced odour generalisation in bees treated with IPA, ASTA and ASTC was evident also from the distribution of responses of individual bees within each group (Fig 2C), which was significantly affected by treatment (χ^2^ = 34.14, df = 12, p<0.001). Amongst controls, the most frequent responses were CS-specific (44%); only 35% of controls responded to one or both of the novel odours tested in addition to the CS (odour generalisation). IPA-treated bees showed higher levels of odour generalisation (45%) than controls (35%), as did bees treated with ASTA (62%) or ASTC (60%), but not ASTCC (38%). Increased generalisation was apparent irrespective of whether or not bees that responded to the CS in the first conditioning trial were included in the dataset (S2 Fig).

### The shift from Type 1 to Type 2 responses is rapid and transient

**Fig 3:**
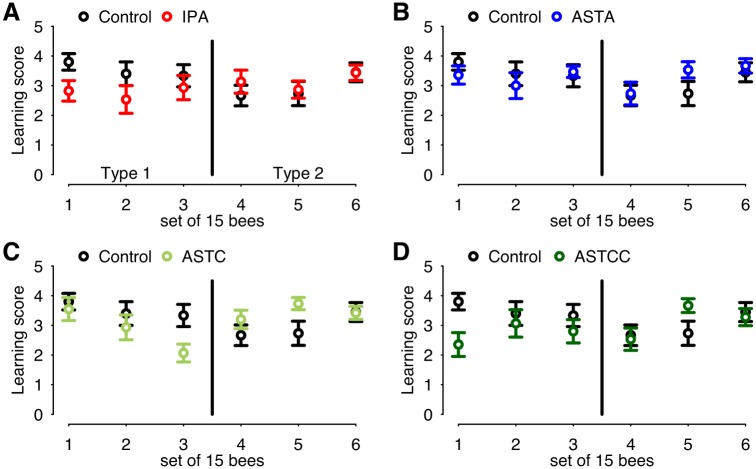
Rapid transition from Type 1 to Type 2 responses. Mean (±SEM) learning scores (the total number of conditioned proboscis extension responses recorded over the 5 conditioning trials) were calculated for sets of 15 bees trained sequentially (sets 1 to 6) over a period of time when bees’ responses to IPA and to allatostatins shifted from Type 1 to Type 2 responses. As all 5 groups were tested in parallel, the same control group appears in all 4 graphs. (**A**) Scores for IPA-treated bees and controls. (**B**) Scores for ASTA-treated bees and controls. (**C**) Scores for ASTC-treated bees and controls. (**D**) Scores for ASTCC-treated bees and controls.

As experiments were conducted on most days of the week, the shift from the more typical (Type 1) responses to Type 2-responses was seen to be rapid and transient, occurring within a period of a few days and lasting approximately 2 to 3 weeks. To examine the transition more closely, the learning scores of bees conditioned on 2 to 3 consecutive days (sets of 15 bees) were averaged during a shift from Type 1 to Type 2 responses. The learning score is the total number of responses observed for each individual bee across the 5 conditioning trials, with scores ranging from 0 to 5. The score values obtained for each treatment group were compared with those of the control group (Fig 3). Interestingly, this approach reveals a tendency for the scores obtained in controls to decline during the shift (time points 1–3 compared to 4–6, W = 1360.5, df = 1, p = 0.07), while scores of treated groups remained stable (IPA W = 1060, df = 1, p = 0.18; ASTA W = 1055.5, df = 1, p = 0.59; ASTC W = 1042.5, df = 1, p = 0.24; ASTCC W = 984.5, df = 1, p = 0.27). Despite some variability across the first 3 time points, bees clearly exhibited Type 1 responses to IPA, ASTC and ASTCC during this period, with controls tending to have higher learning scores than IPA- or AST-treated bees. After the third time point, a shift to Type 2 responses is apparent, where the learning scores of controls are similar to, and sometimes lower than in bees treated with IPA or ASTs.

### Comparing the effects of allatostatins with those of IPA

**Fig 4:**
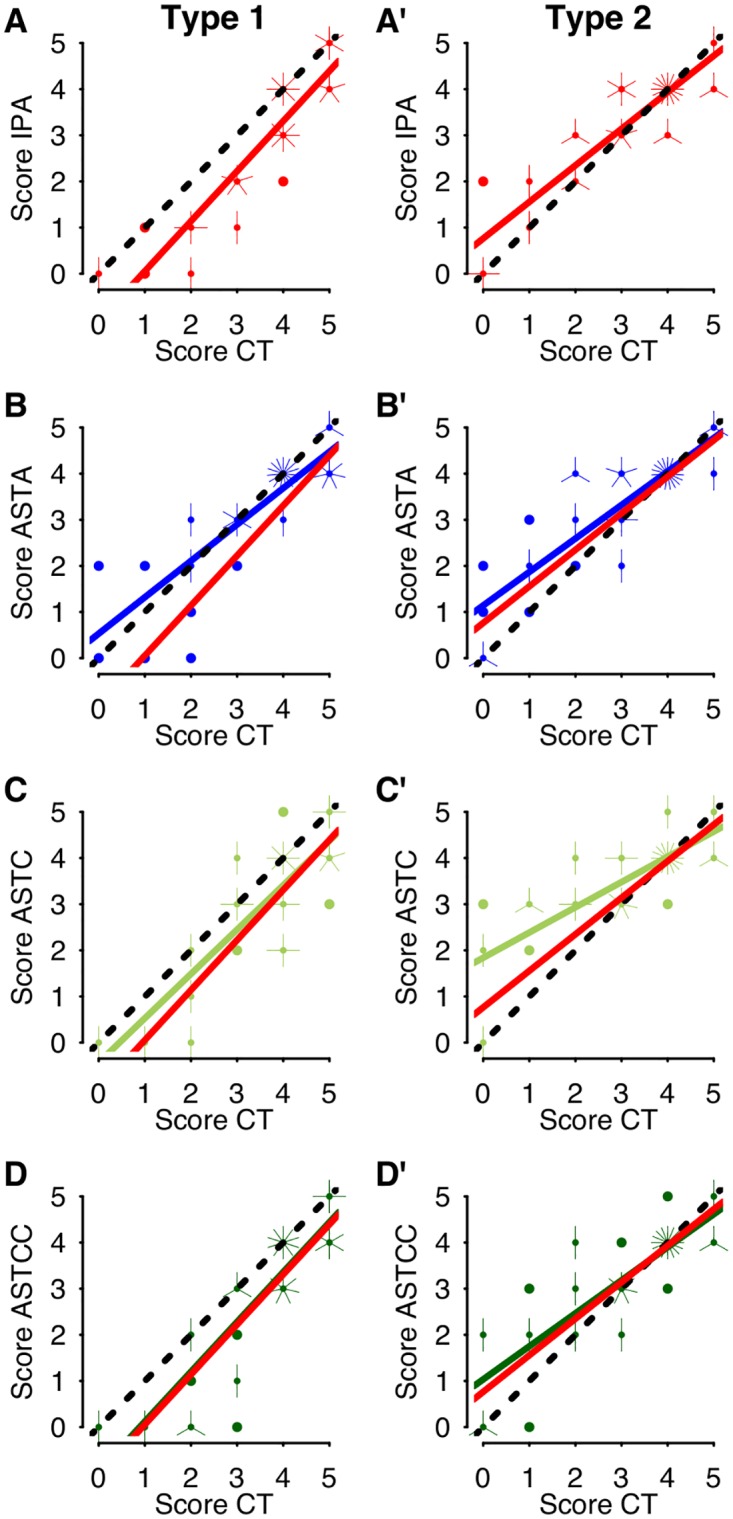
Correlation of learning scores in control and treated bees during Type 1 or Type 2 responses. The learning scores for each individual exposed to IPA (**A**) or treated with ASTA (**B**), ASTC (**C**) or ASTCC (**D**) are compared to those recorded in controls (CT), when bees were showing Type 1 (**A**-**D**) or Type 2 (**A**'-**D**') responses to IPA. The number of branches on each point indicates the number of observations for each score combination. A perfect correlation between the scores of controls and those of IPA- or AST-treated bees would generate points close to the dotted line, with a slope of 1 and slope intercept of zero. Type 1 responses fall below this line, indicating IPA- or AST-induced reduction of learning scores (**A**, **C**, **D**). Type 2 responses fall above this line indicating a shift towards enhanced learning (**A**', **B**', **C**', **D**').

In order to determine which of the 3 ASTs mimics most closely IPA’s effects on learning performance, learning score distributions of treated bees were plotted against those of controls and correlation analysis was used to examine separately the modulatory effects of IPA and of each allatostatin. In bees displaying Type 1 responses to IPA (Fig 4A), the learning scores of IPA-treated bees were lower overall than those of controls, thus shifting the regression line to the right with respect to the line predicted by the null hypothesis of equal performance. In contrast, IPA-treated bees displaying Type 2 responses were less likely than controls to have low learning scores (<3), hence the regression line for this group was deflected to the left (Fig 4A').

The slopes of the regression lines, and slope intercepts (Table 1, top 2 lines), highlight key differences between Type 1 and Type 2 responses to IPA.

**Table 1:** Analysis of correlations of learning scores in controls and treated bees. H0 indicates the null hypothesis being tested. R^2^ represents the adjusted correlation factor of the linear regression. For each comparison, the corresponding Figure number is given. There is a strong correlation between the learning scores of control bees and bees treated with IPA; all groups showed significant learning, as described earlier. In bees displaying Type 1 responses (Fig 4A), the slope of the regression line is not significantly different from that predicted by the null hypothesis, but the regression line intercept reveals that the learning scores of IPA-treated bees displaying Type 1 responses are significantly lower overall than those of controls (CT). In contrast, in bees displaying Type 2 responses (Fig 4A'), the regression line intercept is higher than predicted by the null hypothesis. As low learning scores (<3) were less prevalent in bees displaying Type 2 responses than in Controls, the slope of the regression line deviates significantly from 1. In the lower half of Table 1, the slope and intercept of the regression lines generated for IPA are compared with those generated in the same manner for bees treated with ASTA, ASTC or ASTCC, at times when bees were displaying Type 1 or Type 2 (prime letters) responses to IPA. The analysis shows that effects of ASTCC treatment most closely resemble the effects of IPA.

**Comparison**		**Ho: slope = 1**	**H0: intercept = 0**	**R**^**2**^
**IPA vs CT**	Fig 4A	**1.08±0.07 NS**	**-1.03±0.27** ***	**0.84** ***
**IPA vs CT**	Fig 4A'	**0.80±0.06** ***	**0.76±0.17** ***	**0.81** ***
		**slope = slope IPA**	**intercept = intercept IPA**	
**ASTA**	Fig 4B	**0.79±0.07** **	**0.53±0.27** ***	
**ASTA**	Fig 4B'	**0.73±0.06** ***	**1.14±0.21** *	
**ASTC**	Fig 4C	**0.96±0.10** *	**-0.43±0.36 NS**	
**ASTC**	Fig 4C'	**0.55±0.06** ***	**1.84±0.20** ***	
**ASTCC**	Fig 4D	**1.08±0.09 NS**	**-0.96±0.32 NS**	
**ASTCC**	Fig 4D'	**0.72±0.07 NS**	**1.03±0.23 NS**	

* p<0.05,

** p<0.01,

*** p<0.001,

NS not significant.

When bees were responding to IPA with Type 1 responses, a similar response pattern was observed in ASTC- and ASTCC-treated bees (Fig 4C and 4C', respectively). In contrast, ASTA treatment at this time generated a Type 2-like response (Fig 4B). When bees were responding to IPA with Type 2 responses, ASTA, ASTC and ASTCC treatments all produced Type 2-like responses, with an exaggerated form of a Type 2 response observed in bees treated with ASTC. Comparisons of the slope and intercept of the regression lines generated for IPA with those generated in the same manner for bees treated with ASTA, ASTC or ASTCC (Table 1), reveal that effects of ASTCC treatment most closely resemble the effects of IPA on learning.

### Responsiveness to sucrose and water

**Fig 5:**
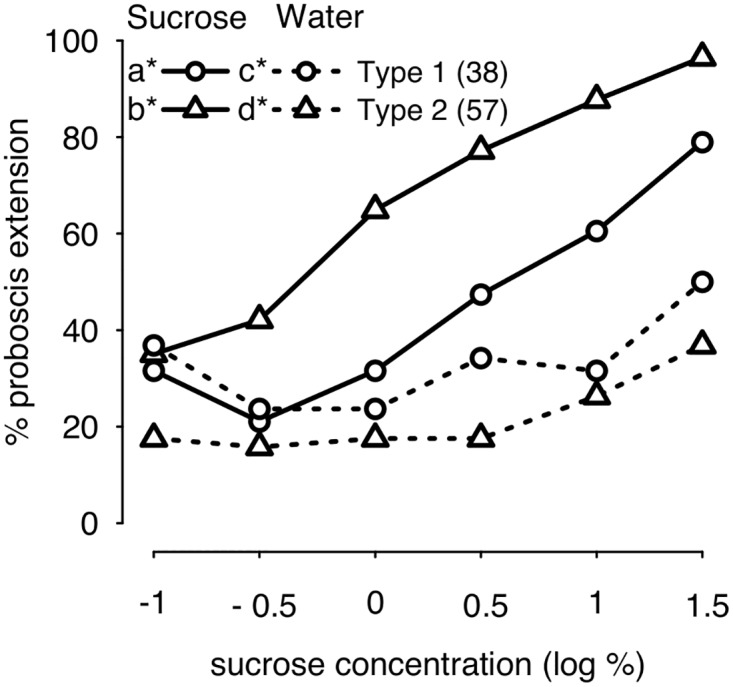
Responsiveness to sucrose and water in controls showing Type 1 or Type 2 responses. Percentages of bees extending their proboscis in response to increasing sucrose concentrations (solid lines) with interspersed water stimulations (dashed lines). Inset: Letters (a, b, c, d) indicate significant differences between groups (refer to text for p values). Asterisk (*) indicates a change in the percentage of bees responding across the 6 presentations of increasing sucrose concentrations or water. The number of bees in each group is indicated in brackets.

As appetitive learning performance is generally positively correlated with a bee’s motivation for sucrose [37–38], we examined the sucrose responsiveness of bees displaying typical (Type 1) versus atypical (Type 2) responses to IPA. Sucrose responsiveness was assessed as described elsewhere [37] by stimulating a bee’s antennae successively with solutions containing increasing concentrations of sucrose, interspersed with water stimulations, and measuring which concentrations elicited proboscis extension. The sucrose responsiveness of bees displaying Type 1 versus Type 2 responses to IPA is presented in Fig 5. The comparisons must be considered indicative only, because bees showing different response types were not tested in parallel. Nonetheless, differences between the two groups are striking. When bees were responding to IPA with Type 2 responses, control bees tested at the same time were more likely to respond to sucrose (1.44±0.66, p<0.05) but less likely to respond to water (-1.74±0.70, p<0.05) than when bees were responding to IPA with Type 1 responses (Fig 5).

## Discussion

Our study reveals that IPA can modulate olfactory learning in two quite distinct ways suggesting that stress responses in bees may be state dependent. In most groups of European bees tested to date, IPA has been shown to slow the rate of acquisition of associative olfactory memories [6–9], and to enhance memory specificity [6]. The present investigation reveals that responses to IPA can shift dramatically in response perhaps, to a change in physiological state. In bees showing atypical (Type 2) responses, acquisition rate tends to be enhanced rather than suppressed by IPA, and in 1-hour memory tests, bees displaying Type-2 responses show odour generalisation, rather than enhanced memory specificity.

Consistent with the hypothesis that stress-related effects of alarm pheromone on learning performance are mediated by allatostatins, we found that when bees’ responses to IPA changed there was a parallel shift in responses to allatostatin treatment. For example, bees responding to IPA with enhanced odour generalisation (a Type 2 response), displayed similar responses when treated with ASTA, ASTC or ASTCC. Bees responding to IPA with reduced acquisition rate and enhanced memory specificity (Type 1 responses), responded similarly to treatments with either ASTC or ASTCC. ASTA failed to inhibit acquisition rate in the present study, but previous work has shown that acquisition can be inhibited also by ASTA [16]. Taken together, these results indicate that stress-related effects of IPA on learning performance in bees could potentially be mediated by ASTA, ASTC and/or ASTCC and perhaps dependent on endogenous levels of these peptides at the time of treatment.

How might allatostatins modulate memory specificity? One possibility in the case of ASTA, is that this neuropeptide modulates the actions of the inhibitory neurotransmitter, GABA. GABA-ergic neurons in primary olfactory centres (antennal lobes) of the honey bee brain have been strongly implicated in odour generalisation. If GABA-mediated synaptic transmission is blocked, for example, odour-induced spatio-temporal activity in the antennal lobes is altered, and odour discrimination in bees is reduced [39–43]. A-type allatostatins are co-expressed in subsets of neurons in the antennal lobes that contain this inhibitory transmitter [26]. Thus ASTA may contribute to shifts towards enhanced or reduced odour discrimination by modulating GABA’s actions on neural circuits in the antennal lobes, and potentially also in areas such as the mushroom bodies of the brain (see [44]).

While there is limited information available, as yet, about the release and actions of allatostatins that co-localise in the insect brain with neurotransmitters, such as GABA [45], activation of allatostatin A-expressing neurons in *Drosophila* has been shown to reduce the fly’s motivation to feed [46]. Recent evidence supports the conclusion also that ASTA regulates metabolism and feeding decisions in the fly [47]. While strongly implicated in the regulation of feeding in several insect species (see reviews by [48–49], ASTs do not appear to affect sucrose responsiveness directly in honey bees [16]. Nonetheless, the apparent change in sucrose responsiveness observed in the present study, associated with the shift from Type 1 to Type 2 responses, suggests a change in arousal, motivation or physiological state. Increases in sucrose responsiveness are generally associated with improved learning performance [38]. However, in the present investigation, higher levels of sucrose responsiveness apparent in bees showing Type 2 responses to IPA and ASTs were associated with enhanced responsiveness to odours, but not with higher learning scores.

The factor or factors responsible for altering bees’ responses both to IPA and to treatment with ASTs have yet to be determined. However, as shown in this study, such shifts can be both rapid and transient. How and where might ASTs be operating? In some, but not all, insect species, ASTs inhibit the synthesis of juvenile hormone (JH) in the *corpora allata* [17]. Whether this is true also of the neuropeptides examined in the present study has yet to be fully explored. While ASTA injection appears not to affect JH biosynthesis in the *corpora allata* of larval honey bees [50], it remains unclear whether this is true also in adults. JH levels in the bee are known to change dramatically with age [51–52] and to contribute to the behavioural development of the bee [53–56], including the development of the stress response to alarm pheromone [57]. In bees of nursing age, stress has been found to increase JH titres [31], as in insects such as *Drosophila melanogaster* [58] and *Manduca sexta* [59]. In older (foraging age) bees, however, effects of stress on JH titres are more variable; foragers with low JH titres show an increase in JH when subjected to stress, whereas in foragers with high JH titres, stress can lead to a decline in JH levels [31]. It is possible that the dual effects of IPA on stress reactivity described in the present investigation represent a behavioural manifestation of the dual effects of stress on JH. The shift to Type 2 responses, for example, could potentially reflect a change in basal levels of JH, or perhaps even of the ASTs themselves. One further possibility is that ASTs modulate the activity of neurons directly involved in associative olfactory learning circuits in the brain of the honey bee. In the fruit fly, for example, ASTA, has recently been shown to enhance appetitive olfactory learning by suppressing the basal activity of a subclass of dopaminergic neurons in the protocerebral anterior medial (PAM) cluster (PAM-γ3-neurons), the activation of which induces aversive memory [60]. This elegant study by Yamagata and colleagues highlights the bidirectional activity of specific dopaminergic neurons and importantly, in the context of the current study, reveals a role for ASTs (specifically ASTA) in modulating neurons that encode behaviourally relevant appetitive and aversive values [60]. It will be exciting in future studies to determine whether ASTs play a similar role(s) in the bee.

## Material and methods

### Animals

In the morning of each day of experiments, forager honey bees *(Apis mellifera)* were collected as they were leaving hives maintained at the Department of Zoology, University of Otago, or departing from colonies in apiaries within the greater Dunedin area (New Zealand). In that case, the local hobbyist beekeeper providing the bees understood that her bees were to be used for the purpose of scientific experiments and authorized us to do so.

Bees were prepared for behavioural experiments immediately after being collected from the hive. The bees were cooled down on ice for a few minutes and then individually harnessed in holders that allowed free movements of the bees’ mouthparts and antennae. After harnessing, foragers were fed with 5 μl of a sucrose solution (50% w/w in water) and the median ocellus was removed as required to enable later injection of allatostatin (or vehicle alone) into the head capsule. All bees were then placed in a dark, warm (25°C) and humidified compartment for 2 hours, before the injection and IPA exposure began (see S2 Fig).

### Allatostatin treatment

The allatostatin peptides, ASTA, ASTC and ASTCC were synthesized by GeneCust (Luxemburg). In the case of ASTA, for which several isoforms exist, we chose to use the form reported to be the most abundant in the bee brain (GRQPYSFGL-amide [18]), and that has been shown to specifically activate the ASTA receptor [16]. ASTC (SYWKQCAFNAVSCF-amide) and ASTCC (GQAKGRVYWRCYFNAVTCF) were both cyclised between their cysteine residues, and they also both specifically activate the ASTC receptor [16]. The vehicle for allatostatins was a saline solution made of phosphate buffered saline (PBS) containing 1mM of the protease blocker, phenylmethanesulfonyl fluoride (PMSF) prepared from a 30 mM stock solution in 100% ethanol. The vehicle, ethanol, was diluted to a final concentration of 3.3%. Control bees were injected with vehicle only (PBS plus 1 mM PMSF).

Allatostatin, or vehicle alone (in controls and IPA-exposed bees), was injected through the median ocellus into the head haemolymph 1 hour before olfactory conditioning (*ie* 2 hours after harnessing and feeding). All injections were performed under a binocular microscope. A micromanipulator-controlled Hamilton syringe was used to inject approximately 200 nl of control or peptide solution (1μM). The dosage of peptide used in this study was chosen based on analysis of effects of dosage undertaken in an earlier study [16]. To make sure that the solution was properly injected, it was pushed out of the syringe so that a drop was visible and then the drop was put in contact with the haemolymph where it was quickly absorbed thanks to the respiratory movements of the bee. Any bee with excessive bleeding was discarded.

### IPA exposure

One hour before the beginning of conditioning, immediately after injection, groups of bees used to examine effects of IPA were exposed to this alarm pheromone component for 30 minutes while the other groups were exposed to vehicle alone (paraffin oil). Harnessed bees were placed individually in a 35 ml glass vial containing a small piece of filter paper soaked with 25μl of IPA (24% in paraffin oil [6]). Controls injected with saline and allatostatin-injected bees were exposed in the same way to paraffin oil alone. The closed vials containing bees being treated with IPA were placed in an air exhaust system throughout the 30 min of exposure period to avoid contamination between groups. Following IPA (or oil) exposure, bees were allowed to recover for 30 min in a dark and humid compartment, before conditioning.

### Olfactory conditioning

Associative conditioning of the proboscis extension reflex was used to monitor effects of allatostatin or IPA treatment on appetitive olfactory learning performance. Odours were delivered to each bee using a 20 ml syringe containing a piece of filter paper soaked with 5μl of pure odorant. To avoid odour contamination, conditioning trials were performed in front of an air exhaust system. Immediately before conditioning, the integrity of the proboscis extension reflex was checked by touching the antennae with a 50% (w/w in water) sucrose solution. Olfactory conditioning of this reflexive response was carried out following a standard protocol consisting of five paired presentations of the odour 1-nonanol (conditioned stimulus, CS) with 50% sucrose (the unconditioned stimulus, US) separated by a 10-min interval. Bees were placed in the learning arena 15 s before the CS was presented, for 4 s. Sucrose was presented 3 s after odour onset, as described by [61], and was delivered first to both antennae to elicit proboscis extension and then to the proboscis. Bees were allowed to lick the sucrose solution for 3 s. Conditioned proboscis extension responses (PER) were defined as extensions of the proboscis further than the mandibular tips that occurred when the odour (CS) was presented, but before the sucrose reward (US) was delivered. Any bee that failed to display reflexive proboscis extension in response to sucrose stimulation of the antennae before, or during conditioning was discarded. Acquisition curves were generated by plotting the percentage of bees responding to the conditioned stimulus at each conditioning trial. In addition, a learning score was calculated for each individual. The learning score is the total number of responses observed across the 5 conditioning trials, with scores ranging from 0 to 5. Individual performances were examined in addition to group performances to help distinguish between differences in numbers of individuals learning, and changes in learning performance [62].

### Memory test

Memory retention was assessed 1 hour after the last conditioning trial. In addition to the CS, 1-nonanol, bees were exposed to 2-hexanol and also to nonanal to determine whether bees were responding selectively to the CS. The two additional odours were chosen according to their degree of perceived similarity with the CS; nonanal is expected to be perceived by bees as being similar to 1-nonanol, whereas 2-hexanol should be perceived as being different [36]. Each bee was placed once again in the learning arena and 15 seconds later the bee was presented with each of the 3 odours in a randomised order, with an inter-stimulus interval of 10 minutes and in the absence of any reinforcement. At the completion of these tests, each bee was stimulated with sucrose to determine whether the reflexive response to sucrose was still intact. Any bee that failed to display the proboscis extension reflex was discarded.

The bees' responses during the memory test were also considered at both group and individual levels. Group responses show the percentage of bees responding to a given odour. To compare the performances of individuals, bees in each group were categorised according to their responses. The responses displayed by bees could theoretically fall into eight distinct categories: bees responding to: (*i*) the conditioned stimulus only (*CS only*), (*ii*) the similar odour only, (*iii*) the dissimilar odour only, (*iv*) the CS plus the similar odour (*CS + similar*), (*v*) the CS plus the dissimilar odour, (*vi*) the similar and the dissimilar odours, (*vii*) the CS, the similar odour and the dissimilar odour (*all odours*), or (*viii*) none of the odours tested (*no response*). However, we found that the responses of 94% of the bees tested fell into one of four response categories; responding to "*CS only*", "*CS + similar*", "*all odours*", or showing "*no response"*. As the remaining categories were represented very infrequently, the response patterns of bees showing atypical responses could not be analysed in any meaningful way. For this reason they were removed from the analysis of individual responses. Individual responses are represented by graphs showing the percentage of bees per group within each of the 4 major response categories.

### Sucrose responsiveness

As appetitive learning performance can be affected by sucrose responsiveness [37–38], responses of bees to sucrose were also examined in this study. Sucrose responsiveness was assessed by stimulating each bee’s antennae successively with solutions containing 0.1%, 0.3%, 1%, 3%, 10% and 30% sucrose, each interspersed with water stimulations in an attempt to limit sensitisation [37]. Typically, a bee that extends her proboscis to a given concentration of sucrose will respond also when tested with sucrose at higher concentrations. If individuals did not answer to a subsequent 50% sucrose stimulation, they were discarded.

### Statistical analysis

The responses of each bee during conditioning and in the memory tests were scored as binary responses (PER was scored as 1, no response as 0). Data were analysed with generalised linear mixed effects modelling (GLMM) using the R package *lme4* [63], using the binomial error structure with the logit-link function, a method recommended for the analysis of categorical data [64]. GLMMs enable comparison of the slopes of the response curves in different treatment groups, with treatment and trial number as fixed factors. Bee identity is used as a random factor. For learning experiments, the fixed factor, trial, was rearranged such that the last trial was set to zero, thus becoming the intercept of the model. In this way, model coefficients represent the difference in slopes between a reference group (usually the control) and a given treatment. Results are presented as coefficients ± standard error.

Appetitive learning was analysed with GLMM to investigate the fixed effects of trial and treatment on responses. No interaction was included, as it did not improve the model. Appetitive memory tests were also analysed with GLMM using odour identity and treatment as fixed factors. Interaction between these two factors was included as it greatly improved the model. Distributions of responses across the 4 response categories were compared using Chi-square tests. Where necessary, Bonferroni corrections were used to correct for multiple comparisons.

Sucrose responsiveness was analysed also with GLMM, using concentration and treatment as fixed factors, as well as their interaction. Bee identities, as well as session identity (sucrose or water) were included as random factors.

To compare the effects on learning performance of IPA, ASTA, ASTC and ASTCC, a learning score was calculated for each bee as the sum of conditioned responses recorded for the 5 conditioning trials. Thus, scores ranged from 0 (no responses recorded) to 5 (a response at each conditioning trial). Within each group, the number of bees displaying each of the 5 scores was recorded. Scores were compared between time periods (see Results section) with Wilcoxon tests. With the scores arranged in ascending order, the distribution of learning scores obtained for each treatment group was plotted against the distribution of learning scores obtained for the control group. Linear regression analysis was used (i) to compare correlations between the learning score distributions of control bees and IPA-treated bees at times when bees were responding with Type 1 versus Type 2 responses, and (ii) to compare at the same time points and in the same way, the effects of ASTA, ASTC and ASTCC. To compare effects of IPA exposure with effects of AST treatment, the slope and intercept point of the regression line obtained following IPA exposure were compared with those of regression lines obtained following treatments with ASTA, ASTC or ASTCC.

## Supporting information

S1 Fig Type 1 responses are conserved when removing bees with spontaneous responses to the CS. (A) Acquisition curves show changes in the percentages of forager bees displaying the conditioned proboscis extension response (PER) over five successive conditioning trials. Inset: key to the 5 groups tested. Letters (a, b, c) indicate significant differences between groups; groups that share a letter are not significantly different. Responses to the CS increased across trials (2.50±0.21, p<0.001). Learning was impaired relative to controls in IPA-exposed (-1.17±0.56, p<0.05) and ASTCC-treated bees (-1.03±0.56, p = 0.06). ASTA and ASTC 689 treatment did not significantly reduce learning rate (ASTA 0.20±0.51, p = 0.70; ASTC -0.63±0.53, p = 0.23). The number of bees in each group is indicated in parentheses. (B) Results of the 1-hour memory test. Response levels across the 5 groups tested do not differ for either of the odours. No differences were observed in response to the CS (IPA 1.72±6.44, p = 0.79; ASTA 1.29±6.75, p = 0.85; ASTC 0.61±5.37, p = 0.91, ASTCC 3.35±5.34, p = 0.53), the similar odour (IPA -0.73±1.15, p = 0.52; ASTA 0.89±1.10, p = 0.41; ASTC 0.34±1.08, p = 0.75, ASTCC -1.49±1.27, p = 0.24), or the dissimilar odour (IPA -4.39±3.10, p = 0.15; ASTA 2.82±2.59, p = 0.28; ASTC -0.76±2.73, p = 0.78, ASTCC -3.99±3.50, p = 0.25). (C) Categorisation of responses during the memory test. No significant differences were detected between the distribution of control (CT) and IPA- or AST-treated bees into the main different response categories (CS alone, CS plus the similar odour, all 3 odours, none of the odours tested; χ2 = 13.17, df = 12, p = 0.36). The number of included bees in each group is indicated at the bottom of each bar. Small numbers of bees showing very unusual response patterns were excluded from the statistical analysis.
(TIFF)

S2 Fig Timing of the experimental protocol. (PDF)
