# Supplementary figures and images for: C-type allatostatins mimic stress-related effects of alarm pheromone on honey bee learning and memory recall
Source: PLoS One. 2017 Mar 21;12(3):e0174321. doi: 10.1371/journal.pone.0174321 (PMC5360335; doi:10.1371/journal.pone.0174321)

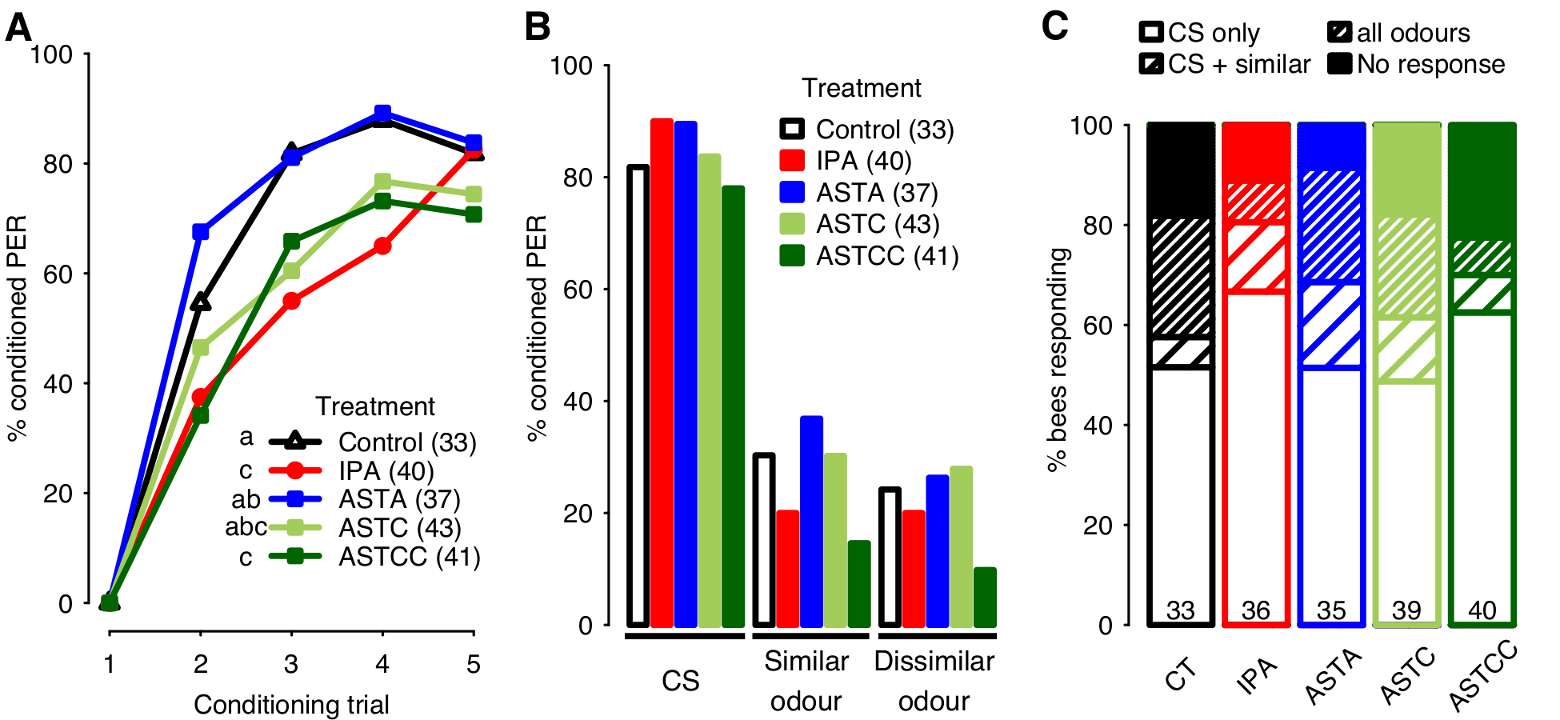

Supplement: S1 Fig — (A) Acquisition curves show changes in the percentages of forager bees displaying the conditioned proboscis extension response (PER) over five successive conditioning trials. Inset: key to the 5 groups tested. Letters (a, b, c) indicate significant differences between groups; groups that share a letter are not significantly different. Responses to the CS increased across trials (2.50±0.21, p<0.001). Learning was impaired relative to controls in IPA-exposed (-1.17±0.56, p<0.05) and ASTCC-treated bees (-1.03±0.56, p = 0.06). ASTA and ASTC 689 treatment did not significantly reduce learning rate (ASTA 0.20±0.51, p = 0.70; ASTC -0.63±0.53, p = 0.23). The number of bees in each group is indicated in parentheses. (B) Results of the 1-hour memory test. Response levels across the 5 groups tested do not differ for either of the odours. No differences were observed in response to the CS (IPA 1.72±6.44, p = 0.79; ASTA 1.29±6.75, p = 0.85; ASTC 0.61±5.37, p = 0.91, ASTCC 3.35±5.34, p = 0.53), the similar odour (IPA -0.73±1.15, p = 0.52; ASTA 0.89±1.10, p = 0.41; ASTC 0.34±1.08, p = 0.75, ASTCC -1.49±1.27, p = 0.24), or the dissimilar odour (IPA -4.39±3.10, p = 0.15; ASTA 2.82±2.59, p = 0.28; ASTC -0.76±2.73, p = 0.78, ASTCC -3.99±3.50, p = 0.25). (C) Categorisation of responses during the memory test. No significant differences were detected between the distribution of control (CT) and IPA- or AST-treated bees into the main different response categories (CS alone, CS plus the similar odour, all 3 odours, none of the odours tested; χ2 = 13.17, df = 12, p = 0.36). The number of included bees in each group is indicated at the bottom of each bar. Small numbers of bees showing very unusual response patterns were excluded from the statistical analysis. (TIFF) [file pone.0174321.s001.tiff]
