# Supplementary material for: C-type allatostatins mimic stress-related effects of alarm pheromone on honey bee learning and memory recall
Source: PLoS One. 2017 Mar 21;12(3):e0174321. doi: 10.1371/journal.pone.0174321 (PMC5360335; doi:10.1371/journal.pone.0174321)

0:00
Harness
Remove ocellus
Feed

1:55
Injection
Saline
ASTA
ASTC
ASTCC

2:00 to 2:30
Control or
IPA exposure

3:00 to 3:50
Appetitive olfactory conditioning
CS 1-nonanol

4:50
Memory test
CS
Similar odour
Different odour

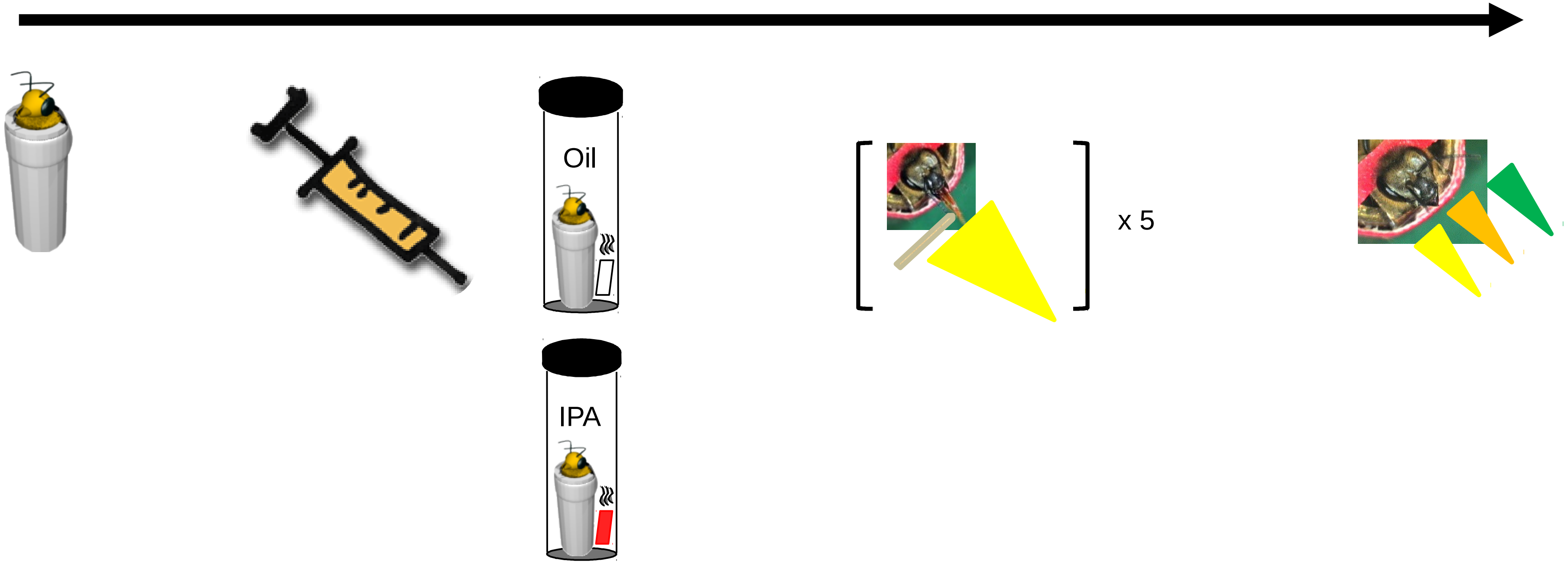

Supplement: S2 Fig — (PDF) [file pone.0174321.s002.pdf]
